# Virtual drug testing: redefining sample collection in a global pandemic

**DOI:** 10.4155/bio-2020-0119

**Published:** 2020-06-12

**Authors:** Matthew N Fedoruk

**Affiliations:** 1 United States Anti-Doping Agency (USADA), Colorado Springs, CO 80919-9918, USA

**Keywords:** anti-doping, DBS, doping, dried blood spots, drug testing, microsampling, sport, tampering, virtual drug testing

## Protecting the integrity of sport & the doping control process

The recent global COVID-19 pandemic is an unprecedented global health crisis with far-reaching impacts on sport. With world sporting events canceled or postponed to limit the spread of infectious disease among athletes and the public and athletes adapting their training programs within the safety and protection of their own homes, global anti-doping programs face the challenge of protecting the health and wellbeing of athletes and sample collection personnel while continuing to pursue the detection and deterrence value of anti-doping testing programs. These uncertain and exceptional circumstances require innovation and flexibility within the current anti-doping system to protect the rights of clean athletes and the integrity of sport.

The World Anti-Doping Code governs global anti-doping programs across Olympic and Paralympic sport. This aims to achieve global standards and harmonization of the operational aspects of anti-doping programs including sample collection and analysis, the results management process, protection of privacy and personal information, the list of prohibited substances and methods, the Therapeutic Use Exemption process, the athlete whereabouts system, intelligence and investigations practices and Code signatories’ rights and responsibilities with code compliance. Within the International Standard for Testing and Investigations (ISTI), specific details with respect to the process of sample collection must be strictly followed to uphold the integrity of the sample and the chain of custody [1]. Some important aspects of the sample collection process, designed to minimize the likelihood of sample manipulation, tampering and to prevent athletes from exploiting loopholes in the system include no-advance notice testing, physically present sample collectors performing in-person sample collection with directly observed urine and blood sample provision and the need for tamper-evident sample collection equipment that ensures the security, suitability and chain of custody of the samples from the time of collection, until laboratory receipt and analysis. Compliance with the current sample collection standards and procedures protect athletes, as well as ensuring that legal challenges to the sample collection process are minimized in cases of a positive test.

As addressed in the current ISTI, witnessing and verifying the provision of the sample is critical to prevent tampering and any attempt to falsify a urine specimen. There have been instances of athletes attempting to use commercially available penile prostheses, urine substitutes, chemical additives that detoxify the sample or attempt to interfere with chemical testing and warming devices to maintain the sample at body temperature [2]. Several commercially available technologies have been developed that can be used to alleviate the invasive nature of a witnessed urine collection that can also identify urine as coming from a specific athlete. One solution is PEGs given orally prior to urination and consequently urine samples can be traced to the donor by analysis of the known urinary PEGs previously ingested [3]. Practical limitations to this approach include requiring elite athletes to ingest a foreign substance and the delay between ingestion and urinary excretion of the PEGs.

## Virtual drug testing as an innovative alternative to complement traditional in-person doping control

Virtual sport drug testing via remote sample collection personnel has been piloted by the United States Anti-Doping Agency (USADA) in order to maintain strict physical distancing and protect the health of elite Olympic and Paralympic athletes. Remote telehealth solutions are similar initiatives which are proven and widely accepted means for delivering virtual medical, health and education services. Recent advances in sample collection technology, combined with technological solutions to reliably and effectively communicate by both video and audio via mobile devices, has enabled USADA to design a novel ‘do-it-yourself’ sample collection program, which allows for both urine and blood sample collection remotely, while upholding the integrity and validity of the sample collection process within the current framework of the anti-doping rules. The elite athletes participating in the pilot program are familiar with USADA and international sport anti-doping programs and have well-established urine and blood sample testing histories. Previous steroidal biomarkers in urine samples and hematological parameters in blood samples, respectively, have established an athlete biological passport (ABP) for each athlete. Thus, the longitudinal monitoring of these specific variables is an established and fit-for-purpose method that aims to detect this biological fingerprint of doping.

Similar to the use of clinical biomarkers used to diagnose or monitor disease progression, biomarkers of doping measured or inferred from blood and urine samples are used to establish the use of doping substances or methods [4]. Past cases of sample manipulation have been identified in sports through the use of steroid ABP and DNA-short tandem repeat analysis which have conclusively demonstrated that drug testing samples collected at different sport competitions were identical [5].

Male and female athletes voluntarily participating in the virtual drug testing program were chosen across Olympic and Paralympic summer sport disciplines including swimming, cycling and track and field. The pilot sample collection program includes no-advance notice testing within the athlete’s ‘1-hour window’, which is a daily period of time that the athlete is required to be in a fixed location and available for testing. Athletes agreed to participate in the program for at least an 8-week period and undergo at least three sample collections per month.

At present, routine doping controls largely rely on testing whole blood, serum and urine samples. These multiple matrices allow sample analyses to comprehensively cover the broad chemical diversity of prohibited substances and their metabolites on the World Anti-Doping Agency (WADA) Prohibited List. Once collected from athletes, anonymous samples identified only by a sample code number are shipped to an accredited anti-doping laboratory in accordance with strict transport requirements to maintain sample integrity. It is important to consider the complexity of various aspects of the sampling procedure and analysis when assessing the added value of implementing alternative matrices for sports drug testing. Some important pre-analytical and analytical factors to consider, include sample collection time and cost-effectiveness, as well as intrusiveness and invasiveness of the collection process to the athlete. In addition, sample volume, analyte stability and overall usefulness of the analysis data to drug detection and result interpretation compared with traditional urine and blood collections are important [6]. Recently, dried blood spots (DBS) is one of biological matrices that have emerged as an alternative and complementary matrix for anti-doping testing and analyses [7]. Published literature in the last decade has demonstrated that the analytical assays in DBS are able to detect many prohibited substances and methods across a wide spectrum of categories on the WADA Prohibited List including non-approved substances, anabolic agents, peptide hormones/growth factors/related substances and mimetics (e.g., growth hormone and erythropoietic stimulating agents), β-2-agonists, hormone and metabolic modulators, diuretics and masking agents, stimulants, narcotics, cannabinoids and glucocorticoids and β-blockers [8–12]. Alternative biomarkers to existing ABP blood biomarkers have also been published and present promising alternatives to detecting blood doping including autologous blood transfusion [13]. Further, recent studies have demonstrated that DBS offers additional valuable information to assist in establishing a window of exposure concerning substances prohibited in-competition only. DBS collected concomitantly with routine doping, control urine samples was shown to assist in establishing proof of recent exposure to stimulants and glucocorticoids [14,15] and DNA analysis could also be used to establish an unequivocal identity between an unwitnessed urine sample and an observed blood sample. Further, to prevent tampering with a stored urine sample, the laboratory analysis includes measurement of bacterial degradation markers as well as comparison to previous urinary steroidal biomarkers in all urine samples as part of an established longitudinal ABP.

## Near-painless DBS microsampling to detect performance-enhancing drugs

Numerous microsampling blood devices have been recently developed in the healthcare industry with the goal of replacing traditional venipuncture collections taken by a phlebotomist in a clinical laboratory setting. With billions of blood collections collected annually for the diagnosis of disease and monitoring of health outcomes, at-home microsampling blood collection devices have the potential to revolutionize the healthcare industry. Equally, due to strict pre-analytical blood collection and shipping requirements and the invasiveness of the venipuncture-based blood collection procedure, near painless microsampling devices have the potential to tremendously improve the athlete blood collection experience and drastically reduce sample collection costs and simplify cold-chain blood shipping while preserving the robustness of analytical assays. One example of an US FDA registered product that has shown promise is the TASSO-Sport On-Demand™ push-button device, which is designed to collect and spot the blood in a secure sample pod with four volumetrically-controlled polymer-based dots with a capacity of 20 μl ± 5% CV, respectively [16]. The blood is collected from the skin capillary bed of the upper arm, 1–5 min after the device is placed on the arm through an adhesive and lancet-activated by the user. The resulting DBS sample can be packaged easily and sent directly to the laboratory inexpensively. This procedure allows for a more consistent, secure and expedited DBS sample collection process through a fully integrated medical device compared with traditional finger lancets and DBS cards.

Overcoming the logistical and technical challenges of implementing a remote sample collection program are relatively straightforward but require adaption of the sample collection procedures and specialized training of the sample collection personnel. Video-based and widely available communication tools including Zoom and FaceTime allow the sample collection to be conducted in a virtual secure environment on a mobile phone or laptop computer, while preserving the legal aspects of the doping control process through screen-sharing of the doping control paperwork and digital capture of athlete acknowledgment and signatures. Instantaneous distribution of the completed documents and same day shipping of the athlete samples to the laboratory complete the sample collection process. Athletes are required to follow essentially the same urine sample collection procedures they are accustomed to, with added safeguards to ensure that no concealed urine or unauthorized persons are in the toilet area by showing the sample collection personnel the bathroom via video. Athletes are not asked to video record the sample provision process for privacy reasons. The urine temperature is tested immediately to confirm body temperature using an adhesive externally placed temperature monitoring strip (TheraPak, USA) on the sample collection cup. The provision of the urine is timed, and the athlete is asked to keep the sample in the view of the video camera at all times after provision. The suitability of the sample for analysis is further confirmed through measurement of the specific gravity of the sample through a colorimetric strip (Siemens MultiStix^®^, Siemens Medical Solutions, USA) which have been demonstrated to be reliable and acceptable under the current WADA ISTI. The authenticity of the sample verified through review of the urinary steroid ABP variables after analysis.

## Protecting clean athletes through innovation

The future success of anti-doping detection and deterrence programs depend on responding to the needs of athletes and adapting to the rapid evolution in fit-for-purpose sample collection and analysis technology. Athletes depend on anti-doping programs to uphold their rights as clean competitors and to protect the integrity of sporting competition. Innovative and athlete friendly solutions such as virtual sample collections which reward those with a demonstrated record of anti-doping rule compliance are an effective complementary component of a modern drug testing regimen. Virtual drug testing has the potential to help drug testing authorities respond to the new reality of increased physical health protections and ensure anti-doping remains at the forefront of innovation that will shape the future success of the fight against doping and maintain a level playing field for clean athletes.

## References

[1] World Anti-Doping Agency. International Standard for Testing and Investigations (2020). http://www.wada-ama.org/sites/default/files/resources/files/isti_march2019.pdf

[2] Thevis M, Geyer H, Sigmund G, Schänzer W Sports drug testing: analytical aspects of selected cases of suspected, purported and proven urine manipulation. J. Pharm. Biomed. Anal. 57, 26–32 (2012). 21955645 10.1016/j.jpba.2011.09.002

[3] Elbe AM, Jensen SN, Elsborg P The urine marker test: an alternative approach to supervised urine collection for doping control. Sports Med. 46(1), 15–22 (2016). 26420237 10.1007/s40279-015-0388-6

[4] Sottas PE, Robinson N, Rabin O, Saugy M The athlete biological passport. Clin. Chem. 57(7), 969–976 (2011). 21596947 10.1373/clinchem.2011.162271

[5] Thevis M, Geyer H, Mareck U Detection of manipulation in doping control urine sample collection: a multidisciplinary approach to determine identical urine samples. Anal. Bioanal. Chem. 388(7), 1539–1543 (2007). 17260133 10.1007/s00216-006-1112-z

[6] Thevis M, Geyer H, Tretzel L, Schänzer W Sports drug testing using complementary matrices: advantages and limitations. J. Pharm. Biomed. Anal. 130, 220–230 (2016). 27040951 10.1016/j.jpba.2016.03.055

[7] Henion J, Oliveira RV, Chace DH Microsample analyses via DBS: challenges and opportunities. Bioanalysis 5(20), 2547–2565 (2013). 24138627 10.4155/bio.13.197

[8] Cox HD, Rampton J, Eichner D Quantification of insulin-like growth factor-1 in dried blood spots for detection of growth hormone abuse in sport. Anal. Bioanal. Chem. 405(6), 1949–1958 (2013). 23263515 10.1007/s00216-012-6626-y

[9] Reverter-Branchat G, Ventura R, Ezzel Din M, Mateus J, Pedro C, Segura J Detection of erythropoiesis-stimulating agents in a single dried blood spot. Drug Test. Anal. 10(10), 1496–1507 (2018). 29877055 10.1002/dta.2418

[10] Reverter-Branchat G, Bosch J, Vall J Determination of recent growth hormone abuse using a single dried blood spot. Clin. Chem. 62(10), 1353–1360 (2016). 27507700 10.1373/clinchem.2016.257592

[11] Thomas A, Geyer H, Schänzer W Sensitive determination of prohibited drugs in dried blood spots (DBS) for doping controls by means of a benchtop quadrupole/orbitrap mass spectrometer. Anal. Bioanal. Chem. 403(5), 1279–1289 (2012). 22231507 10.1007/s00216-011-5655-2

[12] Tretzel L, Thomas A, Geyer H Use of dried blood spots in doping control analysis of anabolic steroid esters. J. Pharm. Biomed. Anal. 96, 21–30 (2014). 24713476 10.1016/j.jpba.2014.03.013

[13] Cox HD, Miller GD, Lai A, Cushman D, Eichner D Detection of autologous blood transfusions using a novel dried blood spot method. Drug Test. Anal. 9(11–12), 1713–1720 (2017). 29045074 10.1002/dta.2323

[14] Thevis M, Kuuranne T, Dib J, Thomas A, Geyer H Do dried blood spots (DBS) have the potential to support result management processes in routine sports drug testing? Drug Test. Anal. 12(6), 704–710 (2020). 32180361 10.1002/dta.2790

[15] Ellefsen KN, da Costa JL, Concheiro M Cocaine and metabolite concentrations in DBS and venous blood after controlled intravenous cocaine administration. Bioanalysis 7(16), 2041–2056 (2015). 26327184 10.4155/bio.15.127 PMC4644129

[16] TASSO-Sport (2020). http://www.tassoinc.com/tasso-sport

